# Characterizing, controlling and eliminating residual malaria transmission

**DOI:** 10.1186/1475-2875-13-S1-P53

**Published:** 2014-09-22

**Authors:** Gerry Killeen

**Affiliations:** 1 Liverpool School of Tropical Medicine, Liverpool, UK

## 

Long-lasting insecticidal nets (LLINs) and indoor residual spraying (IRS) interventions can reduce malaria transmission by targeting mosquitoes when they feed upon sleeping humans and/or when they rest inside houses, livestock shelters or other man-made structures. However, many malaria vector species can maintain robust transmission, despite high coverage of LLINs/IRS containing insecticides to which they are physiologically fully susceptible, because they exhibit one or more behaviours that define the biological limits of achievable impact with these interventions: (1) natural or insecticide-induced avoidance of contact with treated surfaces within houses and early exit from them, minimizing exposure hazard of vectors which feed indoors upon humans, (2) feeding upon humans when they are active and unprotected outdoors, attenuating personal protection and any consequent community-wide suppression of transmission, (3) feeding upon animals, minimizing contact with insecticides targeted at humans or houses, (4) resting outdoors, away from insecticide-treated surfaces of nets, walls and roofs. Residual malaria transmission is therefore defined as all forms of transmission that can persist after achieving full population-wide coverage with effective LLIN and/or IRS containing active ingredients to which local vector populations are fully susceptible. Residual transmission is sufficiently intense across most of the tropics to render malaria elimination infeasible without new or improved vector control methods. Many novel or improved vector control strategies to address residual transmission are emerging that either (1) enhance control of adult vectors that enter houses to feed and/or rest by killing, repelling or excluding them, (2) kill or repel adult mosquitoes when they attack people outdoors, (3) kill adult mosquitoes when they attack livestock, (4) kill adult mosquitoes when they feed upon sugar, or (5) kill immature mosquitoes at aquatic habitats. However, none of these options has sufficient supporting evidence to justify full-scale programmatic implementation so concerted investment in their rigorous selection, development and evaluation is required over the coming decade to enable control and, ultimately, elimination of residual malaria transmission. In the meantime, national programmes may assess options for addressing residual transmission under programmatic conditions through exploratory pilot studies with strong monitoring, evaluation and operational research components, similarly to the Onchocerciasis Control Programme.

